# Roles of the Kinase TAK1 in CD40-Mediated Effects on Vascular Oxidative Stress and Neointima Formation after Vascular Injury

**DOI:** 10.1371/journal.pone.0101671

**Published:** 2014-07-22

**Authors:** Zifang Song, Xiaolei Zhu, Rong Jin, Cuiping Wang, Jinchuan Yan, Qichang Zheng, Anil Nanda, D. Neil Granger, Guohong Li

**Affiliations:** 1 Vascular Biology and Stroke Research Laboratory, Department of Neurosurgery, LSU Health Science Center in Shreveport, Shreveport, Louisiana, United States of America; 2 Department of Physiology, LSU Health Science Center in Shreveport, Shreveport, Louisiana, United States of America; 3 Department of General Surgery, Union Hospital, Tongji Medical College, Huazhong University of Science and Technology, Wuhan, China; 4 Department of Cardiology, The Affiliated Hospital of Jiangsu University, Jiangsu, Zhenjiang, China; Northwestern University Feinberg School of Medicine, United States of America

## Abstract

Although TAK1 has been implicated in inflammation and oxidative stress, its roles in vascular smooth muscle cells (VSMCs) and in response to vascular injury have not been investigated. The present study aimed to investigate the role of TAK1 in modulating oxidative stress in VSMCs and its involvement in neointima formation after vascular injury. Double immunostaining reveals that vascular injury induces a robust phosphorylation of TAK1 (Thr187) in the medial VSMCs of injured arteries in wildtype mice, but this effect is blocked in CD40-deficient mice. Upregulation of TAK1 in VSMCs is functionally important, as it is critically involved in pro-oxidative and pro-inflammatory effects on VSMCs and eventual neointima formation. In vivo, pharmacological inhibition of TAK1 with 5Z-7-oxozeaenol blocked the injury-induced phosphorylation of both TAK1 (Thr187) and NF-kB/p65 (Ser536), associated with marked inhibition of superoxide production, 3-nitrotyrosine, and MCP-1 in the injured arteries. Cell culture experiments demonstrated that either siRNA knockdown or 5Z-7-oxozeaenol inhibition of TAK1 significantly attenuated NADPH oxidase activation and superoxide production induced by CD40L/CD40 stimulation. Co-immunoprecipitation experiments indicate that blockade of TAK1 disrupted the CD40L-induced complex formation of p22phox with p47phox, p67phox, or Nox4. Blockade of TAK1 also inhibited CD40L-induced NF-kB activation by modulating IKKα/β and NF-kB p65 phosphorylation and this was related to reduced expression of proinflammatory genes (IL-6, MCP-1 and ICAM-1) in VSMCs. Lastly, treatment with 5Z-7-oxozeaenol attenuated neointimal formation in wire-injured femoral arteries. Our findings demonstrate previously uncharacterized roles of TAK1 in vascular oxidative stress and the contribution to neointima formation after vascular injury.

## Introduction

Transforming growth factor-β (TGF-β)-activated kinase 1 (TAK1), a seine/threonine kinase, was originally identified as a mitogen-activated protein kinase kinase kinase (MAP3K) which can be activated by TGF-β [1]. Recently, TAK1 has been characterized as a key regulator in immune and proinflammatory intracellular signaling pathways [2]–[4]. TAK1 can be activated by diverse proinflammatory stimuli, such as tumor necrosis factor-α (TNFα), interleukin-1 (IL-1), bacterial lipopolysaccharide (LPS), and CD40 ligand (CD40L) [2], [5], [6]. The activated TAK1 in turn mediates intracellular signaling, via downstream nuclear factor kappaB (NF-kB), p38 MAPK, and c-Jun N-terminal kinase, which may drive inflammatory and oxidative responses in a cell-type specific manner [2], [3], [7]. Although the function of TAK1 in immune cells has been studied extensively, its functions in vascular smooth muscle cells (VSMCs) and vascular diseases remain poorly defined.

Excessive production of reactive oxygen species (ROS) is a central mechanism governing pathologic activation of VSMCs and neointima formation in response to arterial injury [8]–[10]. The present study aimed to investigate previously uncharacterized roles of TAK1 in vascular oxidative stress and the response to vascular injury. Our findings provide the first description that TAK1 plays a critical role in mediating oxidative stress and proinflammatory phenotype changes in VSMCs and that contributes importantly to neointima formation after vascular injury.

## Methods

### Animals

All experimental procedures were carried out in accordance with the NIH Guide for the Care and Use of Laboratory Animals and approved by the Animal Care and Use Committee of the Louisiana State University Health Science Center-Shreveport (IACUC approval number: 0819). Male C57BL/6 mice and CD40^−/−^ mice (backcrossed onto a C57BL/6 background for >10 generations) were obtained from The Jackson Laboratory (Bar Harbor, ME). Mice were fed with a Western-type diet (TD 88137; Harlan-Teklad, Madison, WI) containing 21% fat by weight (0.15% cholesterol and 19.5% casein without sodium cholate) starting 2 weeks before vascular injury as described previously [11], [12].

### Wire-induced vascular injury and drug treatment

Wire-induced injury of femoral artery was performed as previously described [13]. Briefly, mice were anesthetized with intraperitoneal administration of ketamine (80 mg/kg body wt; Abbott Laboratories) and xylazine (5 mg/kg body wt; Rompun, Bayer Corp). The left femoral artery and its muscular branch were exposed, and a 0.014′ (0.36 mm) diameter angioplasty guide wire was introduced into the arterial lumen and advanced to the level of the aortic bifurcation and pulled back three times. In some experiments, mice were treated with either 5Z-7-Oxozeaenol (ZOL, 0.5 mg/kg/day, i.p.) (Calbiochem) (a highly selective TAK1 inhibitor) for 6 consecutive days after the surgery or N-Acetyl-L-cysteine (NAC, 10 mg/ml in drinking water) (Sigma) (a widely used ROS scavenger) starting the first day after the surgery until the end of the experiment. At the end of the experiment, mice were euthanized by intraperitoneal injection of 100 mg/kg sodium pentobarbital.

### Tissue harvesting

At the indicated time points after injury, mice were euthanized and vessels were harvested as described previously [11]. For immunofluorescence and DHE staining, intracardiac perfusion with PBS was performed and the injured left and the uninjured right femoral artery were excised and embedded in OCT compound (Tissue-Tek), freshly frozen slides (10-µm-thick sections) prepared, and stored at −80°C pending analysis. For assessing neointima formation, intracardiac perfusion was performed with PBS followed by 4% paraformaldehyde (PFA), the femoral arteries were then harvested and post-fixed with 4% PFA overnight, and the paraffin-embedded sections (5-µm-thick sections) are prepared.

### Morphometric analysis

Seven serial cross-sections (120 µm apart) were prepared and stained with Verhoeff's elastic stain. Histomorphometric analysis was performed by two blinded researchers using Image Pro Plus 5.0 software and the mean of their results was calculated. We measured the lumen area, intimal area, medial area, and total vessel area at each level as described previously [11]. The mean value of neointimal area, intima/media ratio, percentage of luminal stenosis, and total vessel area was calculated.

### Immunofluorescence staining

Double immunofluorescence staining was performed on frozen sections using the following antibodies: phospho-TAK1(Thr187) (1∶50; Abcam), 3-Nitrotyrosine (3-NT, 1∶50; Santa Cruz), phospho-p65(Ser536) (1∶50; Abcam), MCP-1 (1∶250; Santa Cruz), and SMα-actin conjugated with Cy3 (1∶500; Sigma). Isotype-matched antibodies served as negative controls. Sections were incubated with the indicated antibodies overnight at 4°C. Immunoreactions were visualized using Alexa Fluor 488-conjugated secondary antibodies (1∶200; Invitrogen). Mounting medium containing DAPI was then applied. Images were acquired using a fluorescence microscope (Nikon, Japan). For quantitative comparison of the expression of indicated molecules, the percent of the positively double-stained area to the total traced area was determined in triplicate.

### DHE staining

Freshly unfixed frozen sections were incubated with PBS (pH 7.4) at 37°C for 30 min in a humidified chamber and followed by incubation with dihydroethidine (5 µM, DHE, Sigma) at 37°C for 30 min in the dark. The slides were briefly rinsed with PBS and then mounted with VECTASHIELD HardSet Mounting Medium with DAPI (Vector). DHE fluorescence was quantified by automated image analysis using Image Pro Plus 5.0 software. For low-power (X10) images, DHE fluorescence (intensity by area) was measured in the area between the internal elastic lamina and lumen.

### Cell culture

Mice (10-weel old, male) were euthanized by intraperitoneal injection of 100 mg/kg sodium pentobarbital. VSMCs were prepared from thoracic aortic explants as described previously [14]. VSMCs were grown in DMEM supplemented with 10% FBS, 100 U/ml penicillin, and 100 µg/ml streptomycin. Experiments were performed with cells from passages 5 to 10. In all assays, quiescent VSMCs were obtained by incubating cells for 72 hours in serum-free DMEM containing Insulin-Transferrin-Selenium (Cellgro), 0.1 mg/ml of BSA (Sigma), and antibiotics. Different experiments were performed on the VSMCs from multiple independent isolations.

### siRNA transfection

TAK1 expression was silenced using commercial TAK1 siRNA (Santa Cruz, Catalog no. sc-36607) in cultured VSMCs. A nonrelated scrambled siRNA (Catalog no. sc-37007) was used as a negative control. siRNA transfection was performed as previously described [11]. In brief, cells were plated and grown until 70% confluence. TAK1 siRNA or scrambled control siRNA with transfection reagent mixture was added to the serum-free medium. After 8 hours incubation, the medium was replaced with fresh culture medium, and the cells were incubated for an additional 48 hours before performing experiments. The knockdown efficiency was verified by Western blot.

### Measurement of ROS production

Intracellular ROS production was performed by staining with CM-H_2_-DCFDA (Invitrogen). Pretreatment with or without indicated inhibitors, VSMCs were treated with recombinant mouse CD40L (10 µg/ml; R&D Systems) or vehicle for 6 hours. After removing medium and washing cells with PBS, cells were stained with CM-H_2_-DCFDA (5 µmol/L) for 30 min at 37°C in the dark. Fluorescence intensity was measured with a 96-well fluorescence plate reader using excitation and emission wavelengths of 490 nm and 535 nm, respectively. Data were expressed as fluorescence arbitrary units (FAU).

### NADPH oxidase activity assay

NADPH oxidase activity was measured using lucigenin-enhanced chemiluminescence, as previously described [15]. Briefly, cells were collected and homogenized in buffer containing 20 mM KH_2_PO_4_ (pH 7.0), 1x protease mixture inhibitor, 1 mM EGTA, and 0.5 mM phenylmethylsulfonyl fluoride. NADPH oxidase activity was measured in the assay buffer containing 25 mM HEPES (pH 7.4), 120 mM NaCl, 5.9 mM KCl, 1.2 mM MgSO_4_, 1.75 mM CaCl_2_, 11 mM glucose, 0.5 mM EDTA, 100 µM NADPH and 5 µM lucigenin. Reactions were initiated by addition of 20 µL cell lysis containing 50 µg extracted protein. Photon emission in terms of relative light units (RLU) was monitored every 15 seconds for 10 min on a luminometer. The enzyme activity was expressed as RLU per minute per milligram protein.

### NF-κB p65 nuclear translocation

The nuclear translocation of *NF-κB* p65 was examined using immunocytochemical staining. Briefly, VSMCs plated in chamber slides (Lab-Tek II, Thermo) were pretreated with or without 5Z-7-Oxozeaenol (0.2 µmol/L) for 30 min, followed by stimulation with CD40L (10 µg/ml) or vehicle for 1 hour. After fixation and permeabilization, cells were incubated with anti-NF-κB p65 (1∶100, Abcam) overnight at 4°C, followed by incubation with Alexa Fluor 488-conjugated secondary antibody (1∶200, Invitrogen). Nuclei were stained with DAPI. Images were taken using a fluorescence microscope and quantification was performed using Image-Pro Plus software. Three independent experiments were performed. The results were expressed as percentage (mean ± SEM) of NF-κB p65 positive nuclei-stained cells relative to total cells that were counted at least 8 fields per group under 200 magnification.

### Immunoprecipitation and Western blotting

VSMCs were harvested and lysed with RIPA buffer (Cell Signaling) for Western blotting or NP-40 buffer for immunoprecipitation, both supplemented with a protease inhibitor cocktail (Sigma). Following SDS-PAGE, proteins were transferred to nitrocellulose membranes and probed with primary antibodies (from Cell Signaling, unless otherwise indicated) against phospho-TAK1 (Thr187), TAK1, TRAF6 (Santa Cruz), phospho-NF-κB p65 (Ser536), NF-κB p65, phospho-IKKα/β (Ser176/180), phospho-IκBα (Ser32/36), IκBα, Nox4, p22phox, p47phox, p67phox (Santa Cruz), β-actin (Sigma). Immunoreactivity was detected with ECL (Pierce). For immunoprecipitation, proteins were incubated with the antibody against TAK1 or with isotype control IgG and subsequent analyzed by Western blotting using the primary antibody against CD40, TRAF6 and TAK1.

### Real-time quantitative RT-PCR

Total RNA was extracted from treated VSMCs with TRIzol (Invitrogen), following the manufacturer's instructions. Total RNA was reverse transcribed into first strand cDNA by Reverse Transcription System (Promega). The generated cDNAs were used in a real-time PCR with a iQ SYBR Green Supermix (Bio-Rad) in an iCycler Real-Time PCR Detection System (Bio-Rad). Primer sequences were presented in Table S1. HPRT was used as an endogenous control reference. Data were analyzed and quantified using the ΔΔ*C*_t_ method.

### Statistical analysis

Results are presented as mean ± SEM. Experimental data were analyzed by unpaired 2-tailed Student's *t* test or ANOVA followed by Bonferroni multiple-comparison correction. *P*<0.05 was considered statistically significant.

## Results

### Vascular injury induces TAK1 phosphorylation in medial smooth muscle cells

**Figure 1:**
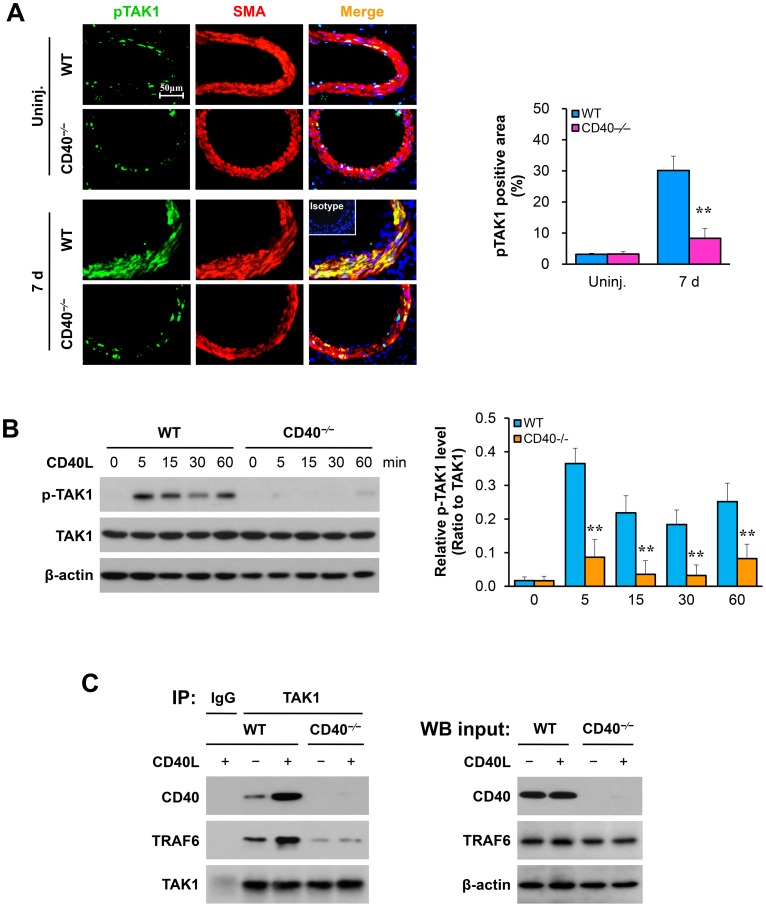
Vascular injury induces TAK1 phosphorylation in the medial smooth muscle cells. (A) Double-staining of pTAK1 with SMα-actin in cross-sections of uninjured (n = 4 per group) and injured (7d) femoral arteries (n = 5 per group) from WT and CD40^−/−^ mice. Inserts show isotype controls combined with DAPI staining. Scale bars: 50 µm. Quantitative analysis of the expression of pTAK1 in media (right panel). **P<0.01 versus corresponding WT group. (B) Western blot analysis of TAK1 (Thr187) phosphorylation in VSMCs stimulated with CD40L (10 µg/ml). The bar graphs represent mean ± SEM values of three independent experiments. **P<0.01 versus corresponding WT group. (C) Co-immunoprecipitation and Western blot show the association of TAK1 with CD40 and TRAF6 in VSMCs. WT and CD40^−/−^ VSMCs were treated with or without CD40L (10 µg/ml) for 5 min. Cell lysates were immunoprecipitated (IP) with anti-TAK1 or mouse IgG, followed by immunoblotting with anti-CD40, anti-TRAF6 or anti-TAK1 (left panel). Whole-cell lysates were immunoblotted with anti-CD40 or anti-TRAF6 (right panel). All results are representative of three independent experiments using the VSMCs from multiple independent isolations.

The expression of TAK1 has not been studied either in injured arteries or cultured VSMCs. Using immunofluorescence staining, we found a low level of the phosphorylated TAK1 (Thr187) in uninjured femoral arteries, but the TAK1 phosphorylation was robustly induced in injured arteries from WT mice, notably with strong co-localization with medial SMα-actin-positive SMCs (Figure 1A). Intriguingly, the injury-induced TAK1 phosphorylation was almost completely abolished in CD40^−/−^ mice (Figure 1A), suggesting that the receptor CD40 is required for the injury-induced TAK1 activation in injured arteries. This possibility is supported by cell culture experiments demonstrating that treatment of VSMCs from WT mice with CD40L robustly induced the phosphorylated TAK1 (Thr187), but failed in CD40-deficient VSMCs (Figure 1B).

The receptor CD40 lacks intrinsic catalytic activity and signals largely through its ability to recruit TNF receptor-associated factors (TRAFs), adapter proteins that bridge receptors of the TNF family to downstream signaling pathways [16], [17]. By performing co-immunoprecipitation experiments, we found that treatment of WT VSMCs with CD40L robustly induced the complex formation of CD40 with both TRAF6 and TAK1, but the TRAF-6-TAK1 complex formation was disrupted in CD40-deficient VSMCs (Figure 1C). These data suggest a previously uncharacterized mechanism of the CD40-TRAF6-TAK1 complex interaction in VSMCs.

### TAK1 inhibition attenuates vascular oxidative stress in response to injury

**Figure 2:**
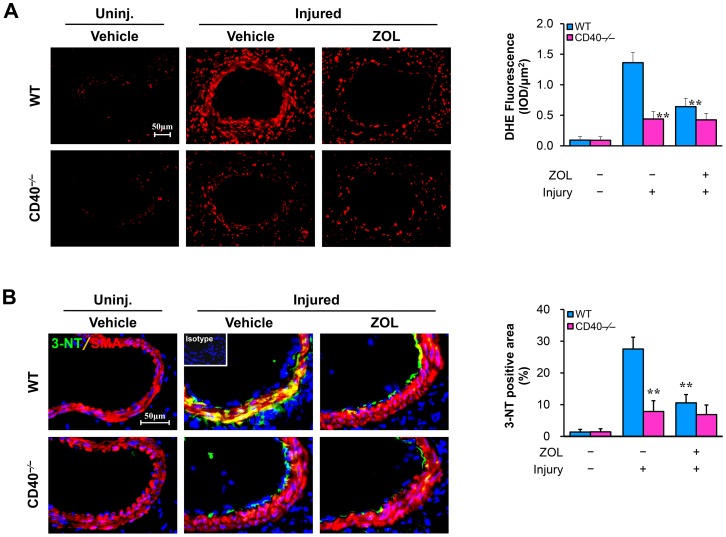
TAK1 inhibition attenuates vascular oxidative stress in response to injury. (A) DHE stained cross sections of WT and CD40^−/−^ femoral arteries from uninjured mice treated with vehicle (n = 5 per group) and injured mice treated with ZOL (0.5 mg/kg/d, i.p.) or vehicle (n = 7 per group) and killed at 7 days after injury. (B) Double-staining of 3-NT (green) with SMα-actin (red) in WT and CD40^−/−^ arteries from uninjured mice treated with vehicle (n = 5 per group) and injured mice treated with ZOL (0.5 mg/kg/d, i.p.) or vehicle (n = 5 per group) and killed at 7 days after the injury. Insert shows appropriate isotype control combined with DAPI staining. Scale bars: 50 µm. **P<0.01 versus injured WT group.

**Figure 3:**
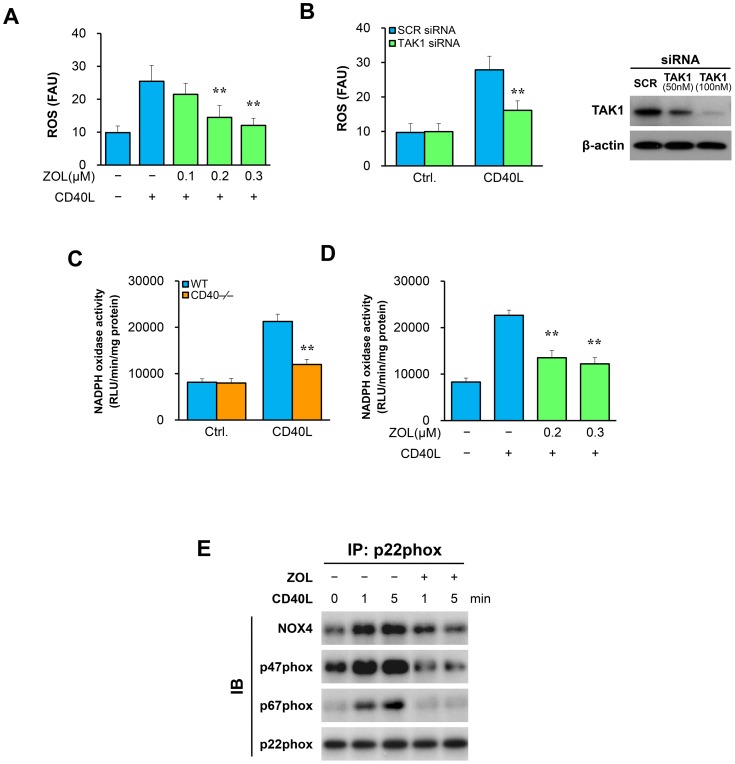
TAK1 inhibition attenuates NADPH oxidase activation in VSMCs. Intracellular ROS levels were assessed by a fluorescence plate reader after staining with CM-H2-DCFDA in VSMCs. Data are expressed as fluorescence arbitrary units (FAU) and represent mean ± SEM of four independent experiments. (**A**) VSMCs pretreated with or without ZOL for 30 min, followed by stimulation with CD40L. **P<0.01 versus CD40L-treated group. (**B**) VSMCs transfected with SCR siRNA or TAK1 siRNA (50 nM) exposed to CD40L. TAK1 levels were assessed by Western blotting in transfected VSMCs (right panel). **P<0.01 versus SCR siRNA group. (**C**) Lucigenin-enhanced chemiluminescence analysis in VSMCs exposed to CD40L for 6 h. **P<0.01 versus WT group. (**D**) Lucigenin-enhanced chemiluminescence analysis in VSMCs pretreated with or without ZOL for 30 min, followed by stimulation with CD40L. **P<0.01 versus CD40L-treated group. (**E**) ZOL inhibits CD40L-induced formation of NADPH complex in VSMCs. VSMCs were pretreated with ZOL (0.2 µmol/L) for 30 min, followed by stimulation with CD40L for the indicated time periods. Cell lysates were immunoprecipitated with anti-p22phox, followed by immunoblotting with anti-NOX4, anti-p47phox, anti-p67phox or anti-p22phox.

Excessive production of reactive oxygen species (ROS) contributes importantly to neointima formation after vascular injury [8], [18]. Using DHE staining to measuring superoxide production, we observed that the intensity of DHE-derived fluorescence (red) was dramatically increased in wire-injured WT arteries, particularly in the medial layer, at 7 days after injury, but, this increase was markedly attenuated in injured arteries from CD40−/− mice (Figure 2A). In vivo treatment of mice with ZOL, a selective inhibitor of TAK1, effectively inhibited the TAK1 (Thr187) phosphorylation and attenuated the injury-induced superoxide production (Figure 2A) and 3-nitrotyrosine (3-NT) (Figure 2B), a marker of oxidative stress, in the medial smooth muscle layers of injured arteries from WT mice. These observations provide the first description that the injury-induced TAK1 activation contributes importantly to vascular oxidative stress in response to tissue injury.

### TAK1 inhibition attenuates NADPH oxidase activation in VSMCs

Next, we investigated the mechanism by which TAK1 mediates ROS production in VSMCs. Using CM-H_2_-DCFDA as a fluorescent probe, we found that CD40L stimulation significantly increased intracellular levels of ROS in WT VSMCs, but this increase was significantly reduced by treatment of VSMCs with a selective TAK1 inhibitor (ZOL) in a dose-dependent manner (Figure 3A). Further, the role of TAK1 in ROS production was verified by siRNA-mediated knockdown of TAK1, which caused a profound inhibition of TAK1 expression in cultured VSMCs (Figure 3B).

The NADPH oxidase represents a major enzymatic source of cellular ROS generation [19], [20]. Here, we investigated whether TAK1 is critically involved in NADPH oxidase activation in VSMCs. Using lucigenin-enhanced chemiluminescence, we measured NADPH oxidase activity in VSMCs. Treatment with CD40L significantly increased NADPH oxidase activity in WT VSMCs, but failed in CD40-deficient VSMCs (Figure 3C). Similarly, inhibition of TAK1 with ZOL significantly attenuated the NADPH oxidase activity in VSMCs stimulated by CD40L (Figure 3D). By performing co-immunoprecipitation experiments, we found that blockage of TAK1 with ZOL disrupted the complex formation of p47phox, p67phox, and Nox 4 with p22phox in VSMCs, that was induced by CD40L (Figure 3E). Previous studies have demonstrated that the Nox4-p22phox complex formation may represent an important mechanism of ROS production in VSMCs [19], [20].

### TAK1 inhibition attenuates NF-κB activation in VSMCs

**Figure 4:**
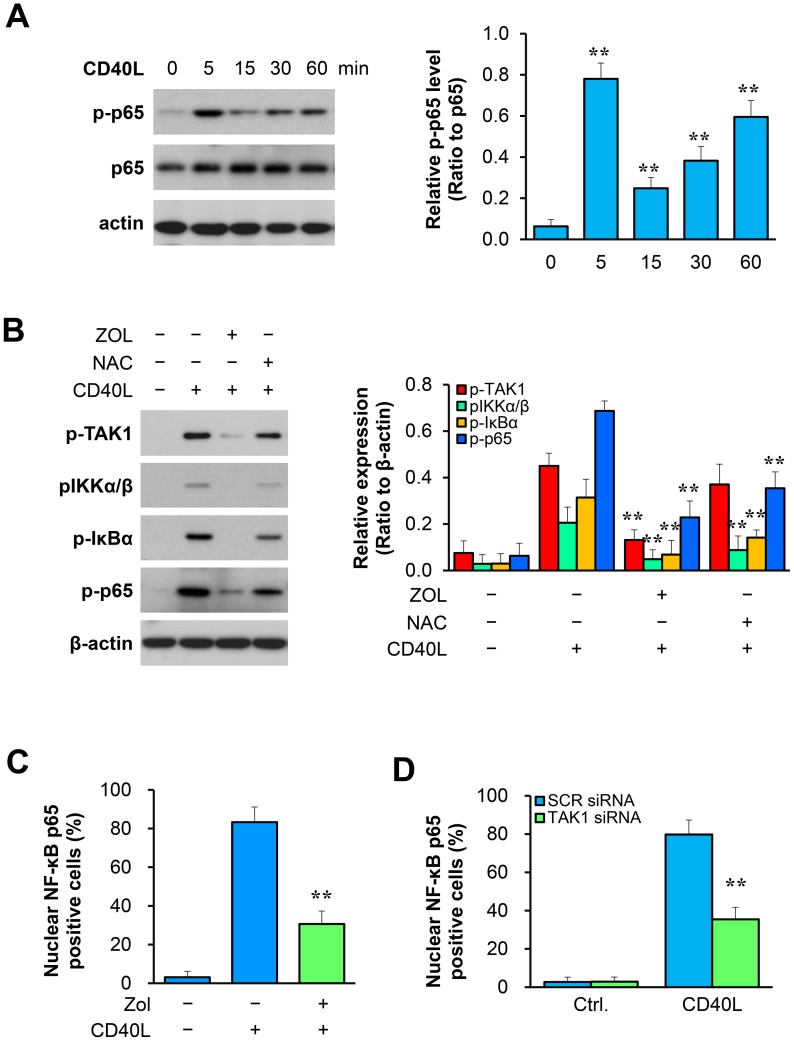
TAK1 inhibition attenuates NF-kB activation in VSMCs. (**A**) Western blot analysis of phosphorylated NF-κB p65 in VSMCs stimulated with CD40L (10 µg/ml). The bar graphs represent mean ± SEM values of three independent experiments. ** *P*<0.01 versus control group. (**B**) Western blot analysis of p-TAK1, p-IKKα/β, p-IκBα, and p-p65 in VSMCs pretreated with ZOL (0.2 µmol/L) or NAC (10 mmol/L) for 30 min, followed by stimulation with CD40L for 5 min. **P<0.01 versus CD40L-treated group. (**C**) NF-κB p65 nuclear translocation was analyzed by immunofluorescence staining in VSMCs pretreated with or without ZOL (0.2 µmol/L) for 30 min and then stimulated with CD40L for 1 h. Quantification of NF-κB nuclear translocation is expressed as the percentage of p65 nuclei-positively stained cells to the total cells. **P<0.01 versus CD40L-treated group. (**D**) Quantitative analysis of NF-κB nuclear translocation in VSMCs transfected with siRNA against TAK1 or with scramble siRNA as a control. **P<0.01 versus CD40L-treated SCR siRNA group.

**Figure 5:**
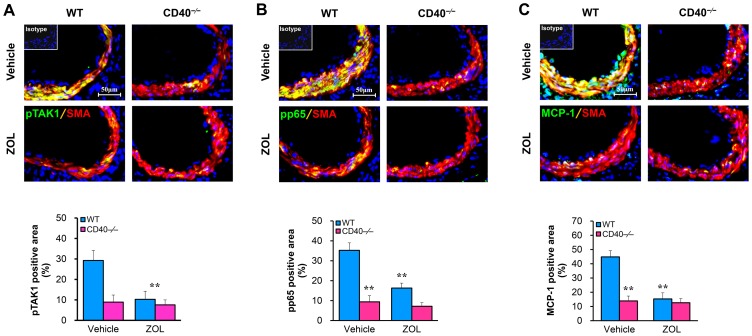
TAK1 inhibition blocks vascular injury-induced phosphorylation of both TAK1 and NF-kB in the vessel wall. WT and CD40^−/−^ mice *were* treated with ZOL (0.5 mg/kg/d, i.p.) or vehicle and killed at 7 days after injury. Double immunostaining shows the co-localization of SMα-actin (red) with (A) phosphorylated TAK1 (Thr187) (green), (B) phosphorylated NFkB-p65 (green), or (C) MCP-1 (green) in the injured arteries, as well as the quantitative analysis (n = 5 per group). Insert shows appropriate isotype control combined with DAPI staining. Scale bars: 50 µm. **P<0.01 versus vehicle-treated WT group.

CD40L stimulated activation of NF-kB in WT VSMCs as shown by a rapid robust increase in p65 phosphorylation (Figure 4A). Pre-treatment of WT VSMCs with the TAK1 inhibitor (ZOL) suppressed the CD40L-induced phosphorylation of IKKα/β at residue Ser176/180, IκBα at residue Ser32/36, and NF-κB p65 at residue Ser536 (Figure 4B). Furthermore, immunocytochemical staining was performed to assess NF-κB p65 nuclear translocation in cultured VSMCs, and data indicate that CD40L-induced p65 nuclear translocation was substantially inhibited by either ZOL inhibition of TAK1 (Figure 4C) or siRNA knockdown of TAK1 (Figure 4D). Consistent with in vitro cell culture data, either ZOL inhibition of TAK1 or CD40 deficiency profoundly attenuated p65 phosphorylation in the medial VSMCs of injured femoral arteries (Figure 5). Moreover, we showed that treatment of VSMCs with ZOL profoundly inhibited the CD40L-induced phosphorylation of IKKα/β, IκBα, and NF-κB p65, but this inhibitory effect was limited in VSMCs treated with the ROS scavenger (NAC) (Figure 4B), suggesting that ROS is less likely to be the major factor mediating TAK1-induced NF-kB activation in VSMCs.

### TAK1 inhibition attenuates proinflammatory phenotype changes in VSMCs

**Figure 6:**
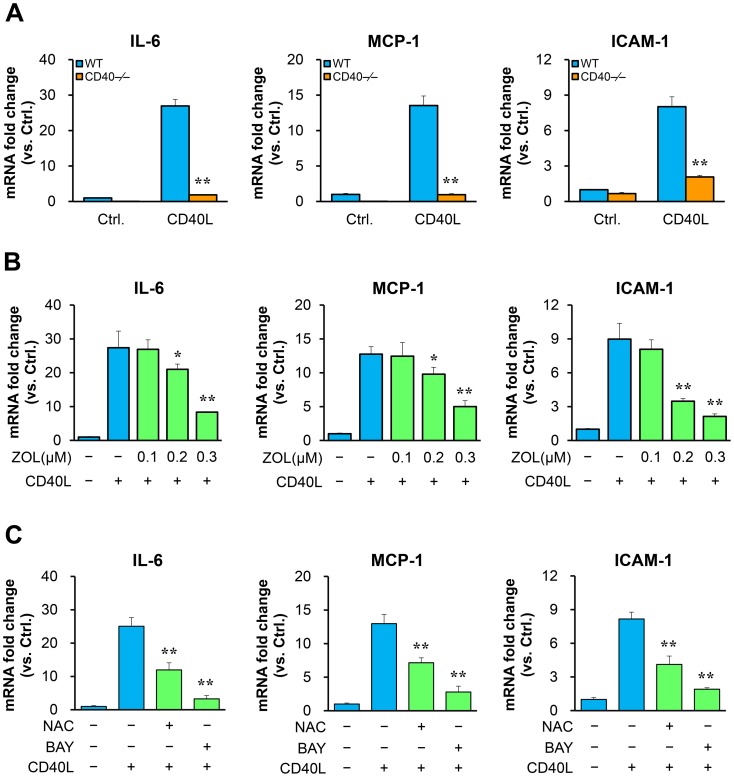
TAK1 inhibition attenuates proinflammatory phenotype changes in VSMCs. mRNA levels of IL-6, MCP-1, and ICAM-1 were determined by quantitative RT-PCR in VSMCs. mRNA levels are normalized to HPRT. (**A**) VSMCs exposed to CD40L (10 µg/ml) for 6 hours. **P<0.01 versus WT group. (**B**) VSMCs pretreated with ZOL or without for 30 min, followed by stimulation with CD40L. **P<0.01 versus CD40L-treated group. (**C**) VSMCs pretreated with the NF-kB inhibitor (BAY, 10 µmol/L) or the ROS scavenger (NAC, 10 mmol/L) for 30 min, followed by stimulation with CD40L. **P<0.01 versus CD40L-treated group. Data are obtained from three independent experiments using the VSMCs from multiple independent isolations.

Treatment with CD40L promoted proinflammatory gene expression, including IL-6, MCP-1 and ICAM-1 in WT VSMCs but failed in CD40-deficient VSMCs (Figure 6A), indicating that the receptor CD40 is required for proinflammatory phenotype changes in VSMCs. Further, we observed that pre-treatment of WT VSMCs with ZOL inhibited the CD40L-induced expression of these proinflammatory genes, in a dose-dependent manner (Figure 6B). Pre-treatment of VSMCs with NAC (a ROS scavenger) or BAY (a NF-kB inhibitor) similarly inhibited the expression of these proinflammatory genes (Figure 6C). In vivo treatment of WT mice with ZOL significantly inhibited the injury-induced NF-kB p65 phosphorylation and MCP-1 in the medial VSMCs of injured arteries (Figure 5).

### TAK1 inhibition attenuates neointima formation after vascular injury

**Figure 7:**
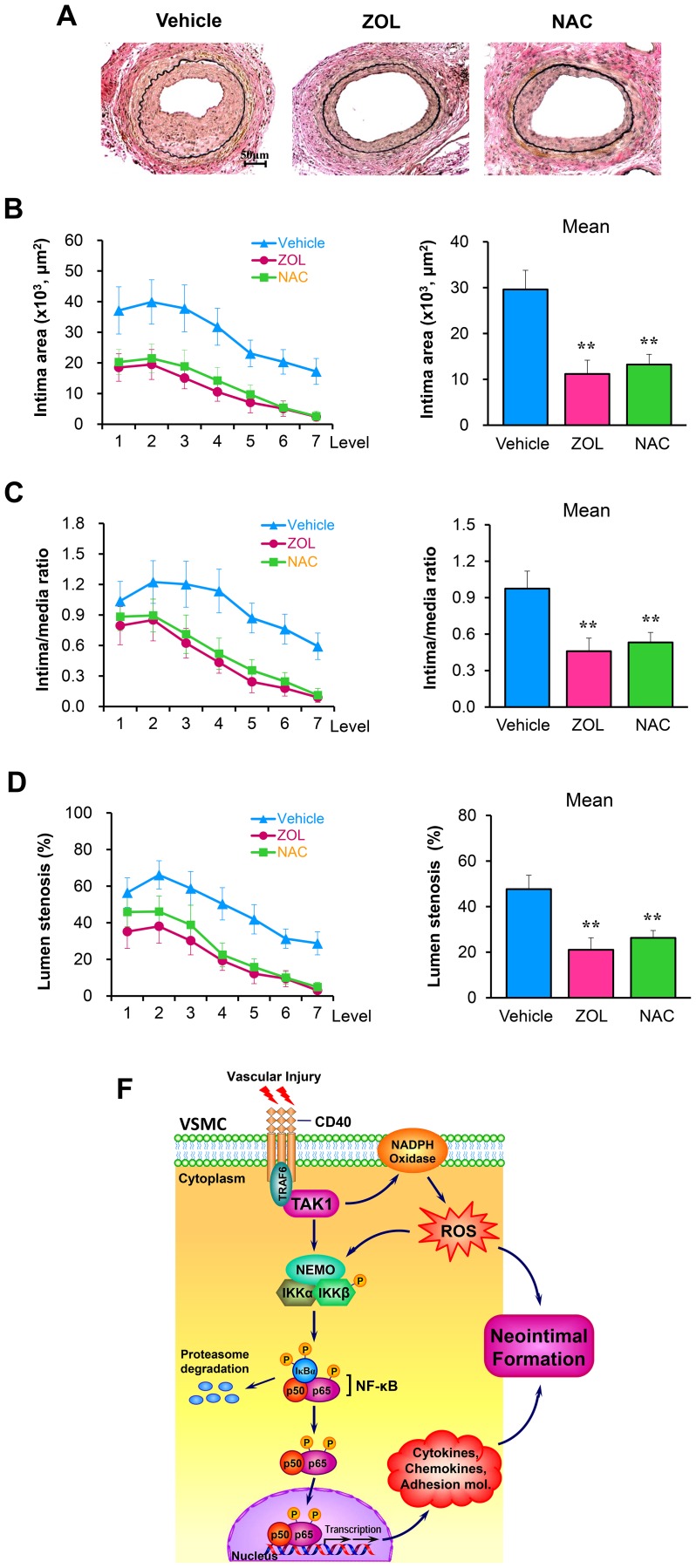
TAK1 inhibition attenuates neointima formation after vascular injury. (**A**) Representative elastic stained cross sections (level 3) of femoral arteries from WT mice treated with ZOL (0.5 5 mg/kg/d, i.p.) (n = 9), N-Acetyl-L-cysteine (NAC, 10 mg/ml in drinking water) (n = 8), or vehicle (n = 8) and killed at 21 d after injury. Scale bars: 50 µm. Intima area (**B**) and vessel area (**E**) were measured at the 7 section levels (120-µm intervals), and the mean area was calculated. Intima/media ratio (**C**) and lumen stenosis (**D**) at each level, and their mean values were determined. **P<0.01 versus vehicle-treated group. (**F**) Schematic model of TAK1 signaling pathway in VSMCs that contributes to vascular inflammation and neointimal formation following vascular injury.

Lastly, we investigated whether TAK1 contributes directly to neointima formation after vascular injury. We found that neointimal formation was significantly attenuated in mice treated with ZOL or NAC (as a control) (Figure 7A). The intima area (Figure 7B), the intima/media ratio (Figure 7C) and the percentage of lumen stenosis (Figure 7D) in injured arteries were comparably reduced between the ZOL- and NAC-treated mice, compared with vehicle-treated mice. In addition, outward vessel remodeling in injured arteries was excluded, as there was no detectable difference in total vessel area among all three groups (Figure 7E). Together, our data suggest that TAK1 contributes importantly to neointimal formation after vascular injury and that may be due to modulating vascular oxidative stress.

## Discussion

In the present study, we provide several novel findings: i) vascular injury induces robust phosphorylation of TAK1 (Thr187) in the injured vessel walls, predominantly in the medial VSMCs; ii) Upregulation of TAK1 phosphorylation in VSMCs appears to play an important role in regulating pro-oxidative and pro-inflammatory effects on VSMCs and eventual neointima formation; and iii) pharmacological inhibition of TAK1 attenuates vascular oxidative stress and reduces neointima formation after vascular injury, suggesting a potential implication of targeting TAK1 signaling in the treatment of vascular disease in the future. These results reveal previously uncharacterized roles of TAK1 in mediating vascular oxidative stress and its involvement in neointima formation in response to vascular injury.

Although TAK1 acts as a key player in inflammatory and immune responses, a clear understanding of TAK1 function in vascular disease is hampered by the fact that TAK1-deficient (TAK1^−/−^) mice displayed severe developmental abnormalities that causes embryonic lethality and defects in vasculogenesis [21], [22]. Thus, TAK1^−/−^ mice are not appropriate model to define the role of TAK1 in the process of vascular injury in vivo. Although recent studies have used the endothelial cell-specific TAK1 knockout mice (TAK^ΔEC^) to show the role of TAK1 in angiogenesis [23], TAK^ΔEC^ mice are not commercially available and are also not suitable for studying the role of TAK1 in VSMCs. In the present study, we used two different approaches, siRNA-mediated gene knockdown technique and pharmacological inhibition of TAK1, to understand the functions of TAK1 in VSMCs and its involvement in neointima formation after vascular injury.

One of the important findings of this study is that TAK1 represents a critical activator in VSMCs to modulate VSMC phenotype to a distinct inflammatory state that help orchestrate local inflammatory response to vascular injury. Inflammation is a critical force driving the initiation and progression of neointima formation following vascular injury [24]–[26]. Mechanical injury that disrupts the vessel architecture triggers the early release of inflammatory cytokines, including IL-1β, IL-6, IL-8, TNF-α, and CD40L, by several cell types including VSMCs that establish autocrine and paracrine signaling loops leading to the development and progression of neointima formation [27]–[29]. Results reported in this study demonstrate that vascular injury elicits early TAK1 activation in the arterial wall, particularly medial SMCs. Although TAK1 has been shown previously to be activated in immune cells by inflammatory cytokines such as TNF-α, IL-1β and CD40L [2]–[6], its activation in VSMCs has not been proven. We demonstrate here that CD40L through engagement with CD40 directly regulates the phosphorylation of TAK1 in cultured VSMCs. In vivo studies have also proven that CD40 deletion abrogates vascular injury-induced TAK1 activation in medial SMCs. In addition, previous work from our group has demonstrated that CD40 signaling via a NF-κB-dependent manner promotes formation of a proinflammatory SMC phenotype characterized by induction of various inflammatory genes including a number of cytokines and adhesion molecules that recruit circulating leukocytes and facilitate vascular inflammation [11]. We now additionally show that the proinflammatory phenotype of VSMCs seen in injured vessel is closely associated with an increased TAK1 activation in the early phase of vascular injury. While previous studies have investigated this concept in different settings, our findings are broadly consistent with the fact that TAK1 plays a pivotal role in regulating proinflammatory signaling pathways in a variety of cell types activated by differing stimuli. Collectively, our findings reveal for the first time that TAK1 is an important mediator of VSMC proinflammatory activation.

Another important finding of our work is the identification of TAK1 as a novel mediator of ROS production in VSMCs. Although the involvement of TAK1 in oxidative stress has been suggested, its contribution to the production of ROS is controversial. It was initially reported that TAK1 deficiency causes elevated ROS production in TNF-stimulated keratinocytes [30]. Consistent with this observation, TAK1-deficient neutrophils produced more ROS in response to LPS stimulation, suggesting that TAK1 is a negative regulator of ROS production [31]. However, in macrophages, TAK1 deletion abolished rather than enhanced LPS-induced ROS production [31]. In this study, we have shown that pharmacological blockade or genetic ablation of TAK1 in VSMCs causes a drastic reduction in CD40-induced NADPH oxidase complex formation and ROS production, defining TAK1 as a positive regulator of ROS production in VSMCs.

Our results also provide novel evidence of oxidative stress involvement in vascular inflammation and lesion progression that is mediated by TAK1 of the VSMCs. Oxidative stress, arising from the damaging action of excess ROS, has been implicated in the pathophysiology of vascular proliferative diseases such as atherosclerosis and neointima formation after angioplasty [8]–[10]. Vascular ROS are produced mainly in medial SMCs in the early phase after vascular injury and derived primarily from NADPH oxidase [8], [32]. It is possible, therefore, that TAK1-mediated neointima formation could be due to its function on ROS production. ROS have been implicated in many aspects of vascular injury, including endothelial dysfunction [33], [34], inflammatory response [10], [35], VSMC proliferation and migration [9], [32], all of which contribute to the development of vascular lesion. However, the intracellular signaling pathways that link ROS to these cellular responses remain unclear. Recently, several studies have suggested that ROS modulate inflammatory response through effects on multiple signal transduction cascades, including MAPK, Akt, and NF-κB signaling [10], [36]. Our study provide further evidence that ROS is responsible, at least in part, for TAK1 induced NF-κB activation that contributes to VSMC proinflammatory phenotype, resulting in vascular inflammation and neointima development after vascular injury.

A question raised by our findings is the precise mechanism by which TAK1 activates NF-κB signaling in VSMCs. A key step for controlling NF-kB activity is the phosphorylation of IκB protein mediated by the IkB kinase (IKK) complex [37]. It has been shown that NF-κB is a redox-sensitive transcription factor [38], [39], and ROS is a crucial player for NF-κB activation in vascular cells in various vascular diseases [40], [41]. Consistent with previous studies, our data implicate the involvement of ROS in TAK1-induced NF-κB activation. We do not know whether ROS induced by TAK1 directly activates IKK. It is possible that some protein kinases would be activated by ROS, thereby mediating the activation of IKK and NF-κB. There are several studies suggesting that IKK is activated by various kinases such as ASK1 and PKD that can be activated by ROS [42]–[44].

Based on the above findings and published results, we propose a working model of TAK1 signaling pathway in VSMCs (Figure 7F). Vascular injury triggers the early release of cytokines that induce TRAF6-TAK1 complex formation, resulting in the activation of TAK1. Activated TAK1 directly phosphorylates IKKβ to activate IKK complex. Alternatively, activated TAK1 may phosphorylates IKKβ and IκBα through inducing the production of ROS. This leads to NF-κB activation and sustained expression of a variety of pro-inflammatory mediators, resulting in vascular inflammation and neointima formation.

In conclusion, we have demonstrated that TAK1 plays a central role in regulating proinflammatory signaling cascades in VSMCs and this regulatory influence of TAK1 is NF-κB-dependent. We also provide evidence that TAK1 mediates NADPH oxidase activation, leading to the production of ROS that contributes to increased IKK phosphorylation and NF-κB activation, as well as the production of inflammatory mediators. ROS appear to act as a key signaling molecule that underlies the neointimal formation following vascular injury. Thus, these findings identify a novel positive role for TAK1 in the regulation of pro-oxidative and proinflammatory signals in VSMCs. This work may have important implications for the development of novel therapeutic approach to treat inflammatory vascular diseases.

## Supporting Information

Table S1 **Primer sequences for RT-PCR.**
(TIF)
