# Supplementary figures and images for: Roles of the Kinase TAK1 in CD40-Mediated Effects on Vascular Oxidative Stress and Neointima Formation after Vascular Injury
Source: PLoS One. 2014 Jul 22;9(7):e101671. doi: 10.1371/journal.pone.0101671 (PMC4106789; doi:10.1371/journal.pone.0101671)

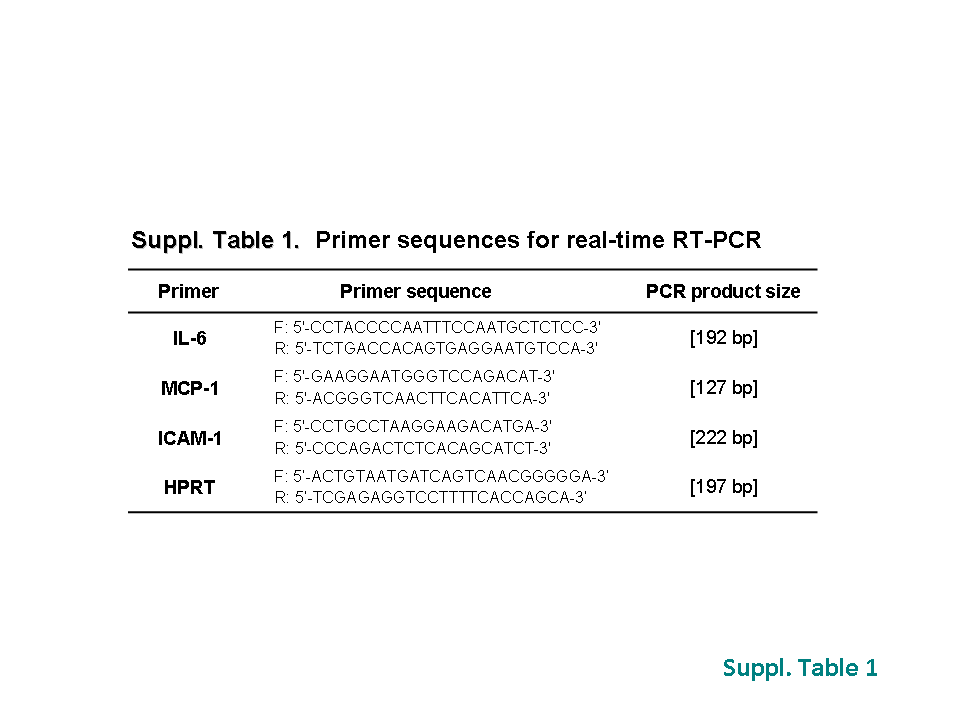

Supplement: Table S1 — Primer sequences for RT-PCR. (TIF) [file pone.0101671.s001.tif]
